# Prolonged Normal Thyroid Function After ^131^I Radioiodine Therapy Using a Minute LT3 Suppression Test (LT3s-RIT) in Patients with Thyroid Unifocal Autonomy and Baseline Detectable TSH

**DOI:** 10.3390/jcm14217871

**Published:** 2025-11-06

**Authors:** Jérôme Clerc, Paul Bodin-Cufi, Louise Giraud, Aurélie Forbes, Emmanuelle Laroche-Masse, Lionel Groussin Rouiller, Louis Schubert, Yvan Mouraeff, Kawtar Hilmy, Anne-Ségolène Cottereau, Eve Piekarski

**Affiliations:** 1 Department of Nuclear Medicine, Cochin Hospital, Assistance Publique Hôpitaux de Paris, University Paris Cité, DMU Imagina, 75014 Paris, Franceeve.piekarski@aphp.fr (E.P.); 2 Department of Nuclear Medicine, Institut du Cancer de Montpellier, Université de Montpellier, 34090 Montpellier, France; 3 Departement of Medical Physics and Radioprotection, Cochin Hospital, Assistance Publique Hôpitaux de Paris, 75014 Paris, France; 4 Department of Endocrinology and Metabolism, Cochin Hospital, Assistance Publique Hôpitaux de Paris, Institut Cochin (U1016 INSERM), University Paris Cité, 75014 Paris, France; 5 Department of Pharmacy, Cochin Hospital, Assistance Publique Hôpitaux de Paris, 75014 Paris, France; 6 Clinical Research Unit, Paris Centre, Assistance Publique Hôpitaux de Paris, 75014 Paris, France

**Keywords:** unifocal autonomy, 131I radionuclide therapy, subclinical hyperthyroidism, thyroid scan, dosimetry, theranostic

## Abstract

**Background**: Subclinical hyperthyroidism grade 1 (SCH G1, TSH > 0.1 mU/L) is common in patients with thyroid unifocal autonomy (UFA) and associated with cardiovascular risks and increased mortality. While ^131^I radioiodine therapy (^131^I-RIT) effectively treats UFA, it frequently induces hypothyroidism, partly due to extra-nodular absorbed dose (AD) enhanced by residual TSH stimulation. **Objective**: We hypothesized that short-term LT3-induced TSH suppression at the time of RIT would promote long-term euthyroidism. **Patients and Methods**: A retrospective study was conducted on 95 UFA patients with SCH G1 (2001–2024). Patients underwent baseline and post-LT3 thyroid scintigraphy, and then received ^131^I-RIT with individualized dosimetry. Long-term bioclinical follow-up was achieved. **Results**: Short-term low-dose LT3 suppression caused no adverse events and significantly reduced TSH (0.45 to 0.047 mU/L). Whole-gland ^123^I uptake decreased moderately (11.0 to 8.4%), while extra-nodular lobe uptake dropped markedly (1.77 to 0.73%) (all *p* < 0.0001). This focused activity on the UFA (2.5-fold increase), maintaining mean UFA AD (about 260 Gy) but reducing extra-nodular AD (61 to 37 Gy, *p* < 0.0001). Despite low ^131^I doses (mean 181 MBq), a dose–response relationship was observed: higher AD correlated with greater nodular lobe volume reduction (*p* < 0.033). At the 88-month follow-up, 93% of patients achieved normal thyroid function; one had persistent SCH G1, two were borderline hypothyroid, and two required LT4. **Conclusions**: ^131^I-RIT under brief LT3-induced TSH suppression induces sustained euthyroidism in SCH G1 with UFA. This simple, low-risk strategy reduces radioprotection concerns and is under evaluation to determine cardiovascular benefits.

## 1. Introduction

The theranostic work-up regarding autonomy due to autonomously functioning thyroid nodules (AFTNs) has evolved considerably over the last 20 years. The routine use of sensitive TSH assays allows for the early detection of mild thyroid hypersecretion, which is currently referred to as subclinical hyperthyroidism (SCH) grade 1 (SCH G1, TSH > 0.1 mU/L) or grade 2 (SCH G2, TSH < 0.1 mu/L) in the presence of normal free plasma thyroid hormone levels [1]. Functional thyroid autonomy is a gradual process that worsens with time [2,3,4,5,6]. Paralleling the increase in functional secretory volume [7], TSH initially fluctuates in the normal range and then slowly decreases to lower-range values. In the present study, we only consider the unifocal autonomy (UFA) variant, excluding multifocal and mixed/disseminated nodular autonomy. Strong evidence indicates that baseline sensitive TSH values are not appropriate for identifying patients with AFTN, with about 50% of patients having normal values. However, a low–normal TSH value may correspond to extra-nodular secretion, such as in disseminated hyperthyroidism harboring hypofunctioning nodules [8,9].

This explains the widely recognized importance of correlative imaging, thyroid scan (TS), and multiparametric ultrasound (MPUS) in identifying patients with thyroid hyperfunction [10,11]. MPUS is the first-line imaging test for exploring a thyroid nodule. It may suggest UFA, but its predictive positive value remains limited, especially in patients with borderline hypersecretion. The only way to ascertain the diagnosis is to obtain a TS that will prove—in the presence of reduced TSH (<0.1 mU/L), either spontaneously or pharmacologically induced by thyroid hormone administration—persistent local hyperfunction in imaging [12,13,14]. UFA, historically referred to as a “hot nodule” [15], presents as a non-suppressible hyper-contrasted nodule, with visible or an absence of visible contrast on the extra-nodular lobe (ENLobe). In SCH G1, ENLobe uptake is more or less activated by residual TSH stimulation, a stage often referred to as compensated nodular hypersecretion [16].

The natural history of the UFA is much better understood. Its evolution with low–normal TSH to toxic variants, SCH G2, and overt hyperthyroidism has been estimated to follow an actual rate of 4–6% in the literature. Since 2006, a large, growing, and convincing body of literature has demonstrated that sustained low thyroid hypersecretion is responsible for a significant rise in cardiovascular morbi-mortality. This mainly involves atrial fibrillation, with actual rates between 5% and 7% a year, especially in patients over 65 years [17,18,19,20,21].

With 70 years of use, radioiodine therapy (^131^I-RIT) has proven to be safe, cheap, easy to administer, and effective in many patients compared with physical methods [16]. The results in eliminating UFA hyperfunction are usually considered good, with cure rates of about 50–75% at 3 months, paralleling a volume reduction of about 35–50% [4,22]. However, long-term data indicate a rising incidence of hypothyroidism with the duration of follow-up. Several factors have been identified to explain this occurrence; in particular, partial iodine uptake suppression of the ENLobe, often due to pretreatment with antithyroid drugs, is regularly reported [16,23].

Because UFA patients diagnosed at an early stage (SCH G1) always have a detectable TSH value, we hypothesized in 1992 that treating compensated UFA patients, after short-term LT3-induced TSH suppression, would limit the occurrence of long-term hypothyroidism while preventing evolution into SCH grade 2. For the first time, we report a new RIT modality in 95 patients with long-term bioclinical follow-up in conjunction with a fine-tuned dosimetric evaluation.

## 2. Patients and Methods

This retrospective study began when we administered ^131^Iodine (^131^I) between August 2001 and January 2024. It took place in a single nuclear medicine center and in a single medical institution (Assistance Publique Hôpitaux de Paris, Paris, France). The current care procedures were used, with a prolonged, spaced follow-up, which is our regular clinical procedure after ^131^I therapy. The present study was endorsed by the ethics committee for Cochin Hospital Publications (CLEP Decision N°: AAA-2023-09044), which declared that “this research was in conformity with the laws and regulations of the country in which the research experiment was performed (Paris, France)”. Informed consent was systematically acquired, stipulating that “the medical, biological and medical data are likely to be used anonymously for the evaluation of care, therapeutic discussions or the production of medical publications.”

The study population comprised 95 patients with SCH G1 due to an autonomously functioning thyroid nodule (AFTN, unifocal autonomy), who were treated with ^131^Iodine after a short (5-day) LT3 administration to suppress TSH.

**Figure 1:**
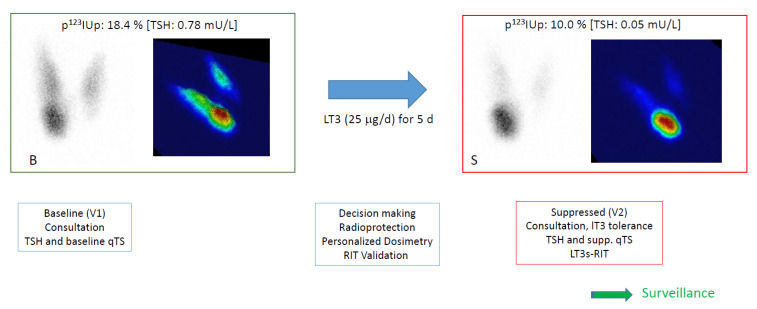
Overview of the protocol. At baseline (B), patients had a first visit (V1), including a consultation and a baseline quantified thyroid scan (qTS) with TSH, often with free T4 (fT4) and free T3 (fT3) sampling. Planar and pseudo-3D images are presented with p^123^I thyroid uptake (p^123^IUp, at 120 min) and the baseline TSH value. Before leaving, the putative procedure was explained, and a T3 ordinance was given to the patient. Decision-making was confirmed with the addressing physician, and treatment was planned and ordered if LT3s-RIT was validated.

The entire procedure requires two outpatient visits, referred to as the baseline procedure (B) and suppressed procedure (S). The global schedule of the procedure is reported in Figure 1.

At the suppressed evaluation (S), the patient would come for a second visit (V2) after a short course of LT3 (at home). They had a suppressed qTS and TSH/fT3 and fT4 sampling in order to assess the diagnosis. The activity was administered, and the surveillance phase was initiated.

### 2.1. Baseline Procedure

Patients presenting with a solitary thyroid nodule and a chronically low TSH value, as assessed by at least 2 previous measurements below 0.60 mU/L in the preceding six months, were referred to our institution to undergo a baseline quantified ^123^I thyroid scan (qTS).

All patients had a recent (<6 months) thyroid ultrasound measuring the volumes of interest, noted on a medical report. We thus used this to record the dimensions or volumes of the extra-nodular lobe (ENLobe), the nodular lobe (NLobe), and the UFA nodule itself. Volumes were calculated according to the ellipsoidal model for one lobe (π/6 × length × width × thickness). If not provided, MPUSs were eventually performed or controlled for in our department, using an Aplio 300 with a linear 18 MHz probe (Canon Medical Systems France SAS, Suresnes 92150, France). Risk of thyroid cancer was ruled out according to guideline recommendations, including MPUS scoring and FNA if necessary [24].

All patients underwent a medical consultation during the baseline procedure to collect a rapid thyroid history, previous imaging, and biological data. Signs and symptoms suggestive of hyperthyroidism were identified, along with the existence of palpitations or tachycardia and the presence or history of atrial fibrillation, other arrhythmic disorders, osteoporosis, and stroke.

All patients underwent a blood test to evaluate thyroid function during the baseline procedure. From 2001 to the end of the study, TSH values were measured with sensitive methods (functional sensitivity < 0.1 mUI/L), allowing us to discriminate patients with normal function (>0.40 mU/L) from those with SCH grade 1 (>0.1 mU/L) or grade 2 (<0.1 mU/L). The last assay used was performed with the CLIA ROCHE kit, with the usual reference range of 0.4–4.00 mUI/L and a sensitivity of 0.04 mUI/L.

All patients had a thyroid scan (TS) during the baseline procedure. The TS was performed after *IV* injection of 6–9 MBq (^123^I), about two hours after injection. We used a gamma camera with a pinhole collimator (6 mm aperture). The digital image was in a 256^2^ format with 70 kcounts or a maximum acquisition time of 900 s (lower uptake values). Before 2022, we used a GE Infinia gamma camera, but more recently, we used one head of an NM/CT 870 DR SPECT-CT (GE Healthcare). Digital TS images were archived on a Carestream PACS (Philips, V 12.1.6.0117) that facilitates post-treatment. All patients had a global thyroid uptake measurement close to two hours post-IV injection, using a thyroid probe (Novelec system, USA Oak Ridge, and more recently, Captus 4000e system from Capintec, USA, New Jersey) with background correction, according to the EANM thyroid uptake guidelines [14]. Actual uptake values acquired at 120 ± 15 min were linearly normalized to 120 min post-injection.

The diagnosis of SCH G1 due to unifocal autonomy relies upon the published criteria for UFA, as reported in the qTS-based classification of hyperthyroidism [25]. This includes both a hyper-contrasted nodule, with a reduced TSH-dependent contrast in the ENLobe, and perturbation of the {Up^123^I/TSH} relation of quantification (see below). We also accepted up to two small (<10 mm) additional autonomous nodules (frontier multifocal).

Demographics and clinical status of our 95 patients at baseline are presented in Table 1. In total, 84.2% had an available dosimetric evaluation at the time of suppression (S), and 76.8% had a fully available dosimetric evaluation (baseline and suppressed). Follow-up information was available in 77% (73/95), with a mean follow-up of 86.1 months (min: 2.2–max: 260.9). Follow-up ultrasounds were available for 52/96 patients (median: 41.7 months).

### 2.2. Treatment Decision-Making

Criteria for treatment proposals were as follows: (1) high suspicion of UFA at the baseline qTS; (2) chronically (n = 3) low TSH levels below 0.60 mU/L, including the TSH sampled at baseline; (3) clinical situations that would benefit from normalization of function (signs or symptoms of hyperthyroidism, rhythmic or ischemic cardiopathy, and nonthyroidal illnesses known to be worsened by hypercatabolism); or (4) cervical discomfort due to nodule size or location. Exclusion criteria included patients with severe evolving cardiac issues, known iodine overload, past history of Graves’ disease or ^131^I administration, and any contraindication to ^131^I therapy. Antithyroid drug therapy (ATD) was not allowed or had to be discontinued for at least 3 months before therapy. According to the precautionary principle, we recommend obtaining feedback from a cardiologist for every patient with rhythmic or ischemic issues at baseline.

The therapy procedure was fully and systematically explained to the patient at the end of the baseline procedure. It included radioprotection handling with a specific form given to the patient and an LT3 prescription (Cynomel^®^, SANOFI Winthrop Industry, 94250 Gentilly, France) at a dose of 25 μg/d for 5 days, including the day of LT3s-RIT.

A medical report was always addressed to the referring physician, with a copy provided to the patient, to validate the therapy plan. Once accepted, the second visit (V2) was planned, a few weeks or months later, to perform the treatment. LT3 suppression had started at home 5 days before V2.

### 2.3. Suppressed Evaluation (S) and Treatment Procedure

On the day of the suppressed evaluation (S) and therapy, we performed a rapid visit to evaluate the tolerance of LT3 suppression and collect any missing data. A new qTS (^123^I) and early uptake measurement were undertaken with TSH blood sampling. Once the diagnosis of UFA was ascertained based on the suppressed TS, we administered the previously planned activity and planned the follow-up.

The therapy to be administered (Aa) was always personalized and planned using the same homemade software. We always used the data from the baseline scan, negating the need for a supplementary visit with the patient. Dosimetry was planned according to the following equation, adapted from the classical Marinelli approach from the guidelines (Equation (1)):

(1) Aa (MBq) = 24.7 × AD(Gy) × target volume (g)/Kmodel ×HL (dy) ×Up 0 (%)

where AD is the intended absorbed dose, HL is the half-life, and Up0 is the extrapolated uptake at time 0 under the hypothesis of single mono-exponential decay of thyroid activity. The chosen target volume was nevertheless modified compared with the usual recommendations [26], where the target mass would be that of the nodule, as determined using ultrasounds. We selected the NLobe volume, an old but robust and clinically relevant determination [27,28], historically referred to as the “dosimetric compromise method”. With this concept, since the NLobe was taken as the whole target, we finally reduced the prescribed dose to 130 Gy, which roughly corresponds to 250–300 Gy regarding the UFA. In patients with larger nodules, we prescribed 200 Gy (about 350–400 Gy to the nodule). Kinetic modeling (Kmodel × HL × Up0) was estimated from large dosimetric databases that were recorded over time in numerous clinical situations.

The administered activities were used a posteriori to estimate the mean absorbed dose (AD) to the NLobe, the ENLobe, the whole gland, and the UFA. This was performed at baseline (B) via simulation (unsuppressed TSH) and at the time of TSH suppression (S). This was partly accomplished with the dosimetric menu of Quanthyr, a new theragnostic software for benign thyroid disease work-up (Planet Oncodose, release 3.3, Dosisoft, Cachan, Fr). Quanthyr combines the thyroid scan image with the ultrasound volumes of interest, adopting an ellipsoidal modeling of the lobes. It relies on conventional dosimetric equations to calculate mean absorbed-dose levels (Gy) in the volumes of interest. In addition it allows calculation of absorbed doses at the pixel level since the pixel depth can be estimated from the ultrasound modeling merged with the thyroid scan image. This finally permits live visualization of the actual isodoses within the gland. For LT3s-RIT, Quanthyr uses an early modified uptake value to predict the actual cumulated uptake, as previously reported. Finally, Quanthyr also provides an algorithmic segmentation of the scan to calculate the volume that retains 90% of the target activity, further referred to as “Metabolic Functional Volume”. We tested whether a higher absorbed dose in the target volume would result in greater volume reduction. We used linear regression and compared mean absorbed-dose levels, producing a 50% or higher volume reduction, looking for an absorbed-dose–effect relationship.

### 2.4. Bioclinical and Follow-Up Ultrasounds

In every ^131^I treatment administered at our department, long-term surveillance is part of our current care procedures. Patients and their physicians were thus asked to plan a specific follow-up, and a protocol form was given to the latter. We recommended a bioclinical follow-up at 3–6 months, 1 year, and 3 years, and then every 3–5 years. Clinical data collection was performed using several methods, including follow-up consultations at the Department of Nuclear Medicine or Endocrinology, consulting the institutional medical database of the Assistance Publique (ORBIS), and requests for information addressed to the attending general practitioners. Biological data included at least a TSH measurement. Follow-up ultrasounds were performed at 1 year and 3 to 5 years thereafter. We always collected the latest ultrasound report available.

Volume variations between baseline data and the last available measurement were calculated for the different volumes of interest as follows (Equation (2)):

(2) VOL variation (%) = 100 × [(last follow-up volume) − (baseline volume)]/(baseline volume)

### 2.5. Statistics

Qualitative variables are described using the number of observations and the frequency of each modality. Quantitative data are described using the medians or means and the SD. Wilcoxon’s test (paired comparison) or the Mann–Whitney test (unpaired comparison) was used. Kaplan–Meier analysis was performed to measure the risk of evolution to SCH grade 2 after LT3s-RIT was compared (rank Mantel–Cox model) with the historical published series of untreated UFA. Logistic regression and sensitivity analysis were performed to identify risk factors for developing hypothyroidism. STATView SAS version 5.0 (SAS Institute Inc. 1992–1998, Cary, NC 27513, USA) was used to carry out the analyses.

## 3. Results

### 3.1. Population

Age at ^131^I-RIT was 53.3 years (mean), a slightly lower value than usual in the literature, as expected for patients with detectable TSH values. The value of TSH to motivate the TS was about 0.35 mU/L, with a 90th-percentile value at 0.56 mU/L. A few patients had slightly higher TSH values, with a strong suspicion of UFA in ultrasounds or cervical discomfort. The UFA was located on the right side in 56.2%, regardless of gender. Thyroid and UFA volumes were slightly higher in men. Though patients were biologically classified as having SCH, 41.0% had detectable signs or symptoms possibly related to hyperthyroidism, mainly palpitations (*n* = 27) and bone loss (*n* = 7). Two had a previous history of stroke.

### 3.2. Suppressed Procedure

Tolerance of LT3 suppression: No significant adverse side effects were evidenced after LT3 administration at low doses (25 μg/d) for a short time (5 days). A few patients with palpitations at baseline still experienced palpitations during the short period of suppression.

**Figure 2:**
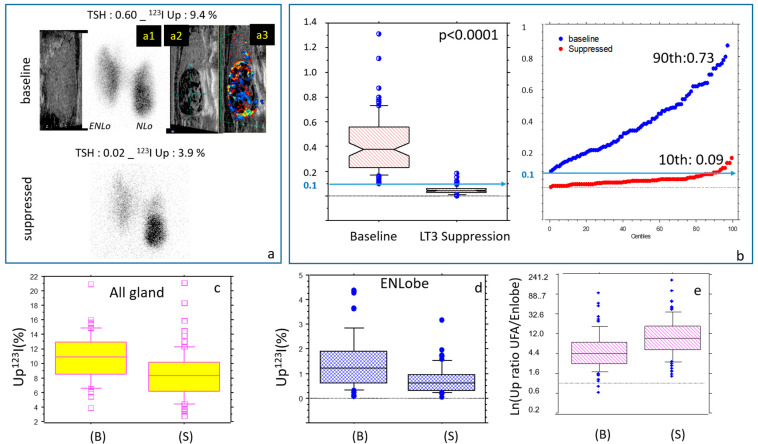
Minute LT3 suppression test (mLT3sT): Effects on the main biological parameters. (**a**) Baseline and suppressed quantified thyroid scan with correlative Multiparametric Ultrasounds (MPUS) suggestive of an AFTN. ENLo: Extra-nodular lobe, NLo: Nodular Lobe. (a1): Thyroid scan image of the NLo; correlative MPUS of the NLo.: (a2): grey scale image and (a3): colored Doppler of the NLo showing increased vascularization of the UFA. Note that the ENLobe displays minor persistent diffuse ^123^I activity at the time of suppression (frontier variant). (**b**) Low-dose LT3 (25 μg/d for 5 d) succeeds in reducing TSH, decreasing the global early uptake (**c**), especially in the ENLobe (**d**), resulting in a strong increase in the uptake ratios (UFA/ENLobe, logarithmic display) (**e**). All variations are significant, *p* < 0.0001).

Effects of LT3 suppression (Table 2 and Figure 2): LT3-induced TSH suppression significantly affected most biological and isotopic parameters (*p* < 0.001).

The short-term suppression proved to be effective in reducing TSH (≤0.1 mU/L) levels in 72.3% of the patients. The remaining patients had borderline suppressed values (0.107–0.150), and one had a value of 0.18. The risk factor for a lack of complete suppression was a baseline TSH of over 0.60 mU/L (*p* = 0.039). Free T4 was not significantly different or slightly decreased, in contrast with a rise in measured free T3 plasma levels sampled at the suppressed qTS.

On a global quantitative level, early ^123^I uptake significantly decreased from about 11% to 8%, in agreement with the non-suppressibility of the nodular uptake.

On a spatial distribution level, overall, the LT3 suppression was highly effective in focusing ^123^I activity onto the UFA and NLobe (autonomous tissue) while suppressing uptake in the ENLobe (TSH-dependent tissue). This was also evidenced by the strong increase (about ×3) in the uptake ratio (UFA/ENLobe) between the baseline and suppressed periods. Finally, we found that the metabolic functional volume was also lowered through suppression (*p* < 0.0001).

### 3.3. Dosimetric Evaluation

According to our two levels of intended absorbed doses (e.g., 130 Gy and 200 Gy), the administered activities significantly differed (*p* < 0.0001). In the low-dose group, patients received (mean ± SD) 146 ± 59 MBq [10th percentile: 76, 90th percentile: 222], while the high-dose group, where an additional volume reduction effect was expected, received the following values: 271 ± 117 MBq [10th: 140, 90th: 374]. UFA volumes were lower in the low-dose versus high-dose group (4.6 ± 2.9 mL versus 8.0 ± 4.9 mL, *p* < 0.0001). Finally, the selected clinical goal was clearly related to the UFA volume at baseline (*p* < 0.0012, OR: 1.27 [1.10–1.47]).

**Figure 3:**
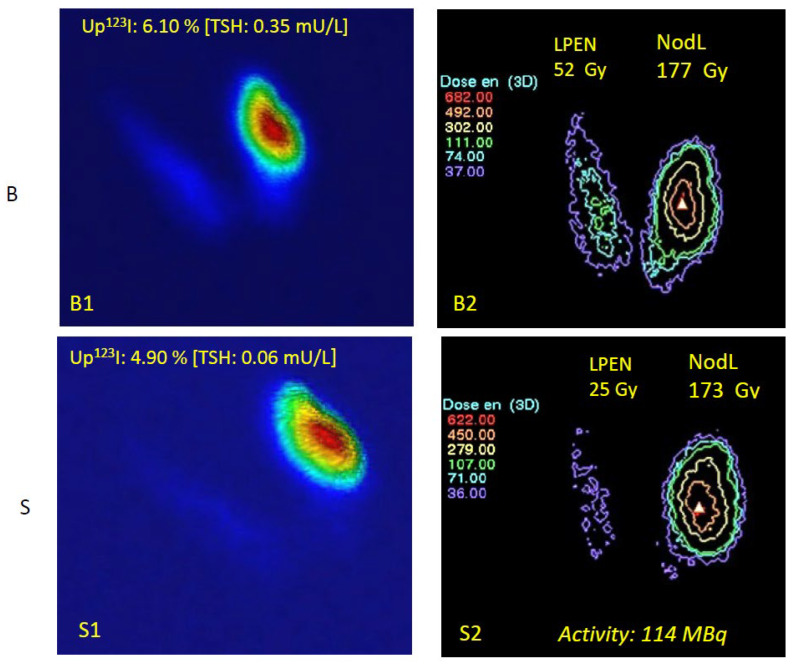
Absorbed doses (ADs) at baseline (B) and under LT3 suppression (S). Baseline: (**upper panel**): (B1) Quantified thyroid scan (pseudo-3D display) and (B2) mean absorbed doses (Gy) if RIT is provided without LT3 suppression. Suppressed (**lower panel**): (S1) Suppressed qTS with lower TSH, lower ^123^I uptake, and (S2) suppressed corresponding AD. The thyroid scan images are displayed using a classical color scale from blue (low activity) to red (high activity). Administered activity: 114 MBq (goal: 130 Gy in the NLobe). The AD applied to the ENLobe decreased from 52 to 25 Gy, a very low value that cannot significantly destroy this normal TSH-dependent tissue. Absorbed doses are displayed using the isodose display menu of Quanthyr (Planet Oncodose, release 3.3, Dosisoft, see Section 2.3). Absorbed dose levels are delineated by the coloured isodoses curves (for instance the green isodose in B2 corresponds to 111 Gy). Note the visible relocalization of the dose in the UFA.

Absorbed doses calculated at baseline for the NLobe were in good agreement with the prescriptions, with a small systematic underdosage of about 10%. As a general rule, actual absorbed doses on LT3 are slightly lower than baseline values, except for the UFA itself, where irradiation levels are preserved during suppression. Notably, 130 Gy in the NLobe (“dosimetric compromise”) corresponds to about 260 Gy in the UFA (anatomical volume). The most important information is that the extra-nodular lobe will benefit from an average 60% dose reduction after LT3 suppression. Changes in absorbed doses at baseline and after LT3 suppression are reported in Table 3 and illustrated in Figure 3.

### 3.4. Follow-Up

At the end of the follow-up, thyroid function was normal except in five patients. The TSH levels were almost always in the normal range (mean ± SD: 1.76 ± 1.18 mU/L, min: 0.26–max: 6.0 mU/L) but significantly higher than baseline values (*p* < 0.0001). Only 5.5% of the patients experienced hypothyroidism under yearly surveillance (mean time of occurrence: 80.7 months, [min: 32.4–max: 118.5]), with two on LT4 substitution, and two others with TSH values of 4.40 and 4.80 mU/L. Only one patient had persistent SCH grade 1 after a 269-month follow-up, with the following biological values: TSH—0.26 mU/L; free T4—15.9 pmol/L; and free T3—4.8 pmol/L. One patient was strongly underdosed and required retreatment with ^131^I. Due to the few patients who experienced biochemical perturbations, it was not possible to individualize any significant risk factor explaining these evolutions. Sensitivity analysis was unsuccessful in identifying a significant risk factor. Even considering a short follow-up (25th: 29.3 months), where no events occurred, the *p* value was still measured at 0.22.

Regarding clinical follow-up, most symptomatic patients were efficiently treated, with the systematic disappearance of palpitations and tachycardia. Three patients underwent surgery. One complained of a persistent cervical discomfort while TSH normalized. Two others were diagnosed as having thyroid cancer, which developed during the follow-up. One had a rare aggressive angiosarcoma of the thyroid at a 188-month follow-up, with a 29 mL nodule. The other developed a new 17 mm nodule with suspicious FNA. Final histology displayed a well-differentiated papillary cancer, while the UFA had reduced to 15 mm with a benign histology.

### 3.5. Absorbed-Dose–Effect Relationship

**Figure 4:**
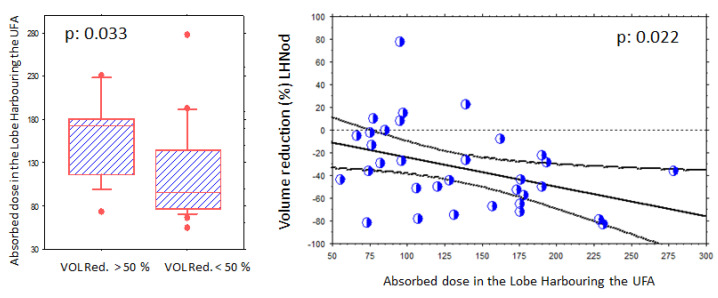
Evidence of a volume reduction absorbed-dose effect relationship in the nodular lobe (NLobe). (**Left panel**): A higher volume reduction (>50%) is observed in patients receiving higher absorbed doses. (**Right panel**): Significant (*p* < 0.022) relationship between volume reduction and absorbed-dose levels in the NLobe. The mean reduction in this lobe was 33.5% (53.2% for the AFTN). Each blue circle shows one volume reduction data (%, Y axis) in relation to an absorbed dose level (Gy, X axis).

Absorbed-dose–effect relationships are illustrated in Figure 4. A higher volume reduction (>50%) was observed when higher absorbed doses were delivered to the NLobe: 157 ± 47 Gy (volume reduction > 50%) versus 118 ± 55 Gy (volume reduction < 50%, *p* = 0.033). However, this was not observed for the UFA itself (*p* = 0.47).

Overall, a significant relationship was observed between volume reduction in the NLobe and the absorbed-dose levels (*p* = 0.022).

UFA volume reduction was 53.2 ± 38.3%, with a lower volume reduction in the NLobe (33.5 ± 33.9%). The ENLobe had a limited mean reduction volume, but demonstrated significant variations among the patients (6.1 ± 59.4%).

## 4. Discussion

Our study reports, for the first time, the effectiveness of ^131^I therapy in UFA patients with SCH G1 using a new RIT protocol, including LT3 suppression of TSH at the time of treatment and a personalized computer-aided theranostic approach (LT3s-RIT). We opted to design LT3s-RIT as a very simple procedure requiring only two outpatient visits. Beyond the clinical relevance of this new approach, it explains why hypothyroidism can be avoided in many patients for years. Indeed, the regional fine-tuned dosimetric evaluation demonstrates reduced irradiation in the normal extra-nodular tissue under TSH suppression. Finally, using two levels of irradiation on the nodular lobe, we demonstrated a volumetric dose–effect relationship that has not been clearly reported before.

Treating SCH patients during an early period is challenging. Indeed, eliminating even mild thyroid hypersecretion should remain a main goal of ^131^I-RIT. In our series, about 40% of the patients were addressed because they complained of signs and symptoms that most often disappeared after treatment. Regardless of the dosimetric protocol used, administering ^131^I to patients with detectable TSH carries a higher risk of causing hypothyroidism as the target volume will, unfortunately, include normal TSH-dependent tissue. This may explain why some doctors are reluctant to treat in the absence of clinical signs, a frequent presentation in subclinical hyperthyroidism.

We achieved our clinical goals based on our hypothesis that treatment with ^131^I under LT3 suppression allowed maintenance of euthyroidism for years in most patients. Spaced but prolonged monitoring is still ongoing for those patients treated with LT3s-RIT to confirm these results in the very long term.

The calculated 0.26%/y rate of progression toward hyperthyroidism in our LT3s-RIT-treated patients favorably compares with spontaneous evolution into SCH G2 or overt hyperthyroidism in untreated UFA. The latter is well documented in the literature [16,29] with a reasonable estimate of 4.1%/y in the largest study [3]. Accordingly, one may calculate a cumulative rate of about 22% from this historical series as compared to less than 1.5% in our patients (*p* < 0.001).

Regarding SCH clinical issues, excellent reviews fully describe the detrimental consequences of chronic mild thyroid hypersecretion, including atrial fibrillation (AF), strokes, heart failure, possibly bone loss, and very frequently symptomatic signs that affect quality of life [17,18,19,21,30]. In a recent large-scale study by Selmer et al., AF had an incidence rate ratio of 1.16 compared with that of normal subjects. If it is true that AF occurrence rises with age, it may be due to increased cardiovascular comorbidity, while in younger adults, SCH is often a major cause of AF [31].

Although uncommonly conducted, recent therapeutic intervention studies suggest that treating SCH patients is clinically relevant. L. Hegedus’s group reported that the hazard ratio for mortality is increased in untreated (×1.23) but not in treated hyperthyroid patients. This was also confirmed in the SCH group, since adjusted mortality HRs for treated versus control patients were 0.87 vs. 1.36, respectively. This suggests that an early therapeutic intervention would be beneficial [20]. They also demonstrated that RIT in postmenopausal women with SCH prevents bone loss for at least 2 years, though this was evaluated at 2% per year without treatment [32]. The most interesting endpoint is the potential preventive effect of RIT on the most serious events: AF, strokes, and death. A recently published randomized study by B. Goichot et al. on 144 SCH G1 patients found, for the first time, that the risk of AF was significantly reduced in patients with normalized TSH, regardless of age and baseline SCH grading [33]. All these recent findings are strong evidence arguing for an earlier work-up and treatment of SCH G1. However, to do so requires practitioners to prove that an early therapeutic intervention will not produce unwanted side effects, notably hypothyroidism.

Notably, our LT3s-RIT approach has a very low incidence of hypothyroidism and a high rate of TSH normalization (95%), even when using severe criteria and in the long term. Considering the results of the excellent study by C. Ceccarelli, a much higher predicted hazard is evident for hypothyroidism, reaching about 80% for a similar follow-up duration in patients with TSH-dependent partial uptake suppression in the ENLobe [23]. In the same vein, an undesirable 25% of patients developed hypothyroidism in Goichot’s study [33].

Indeed, among the well-established risk factors for hypothyroidism among duration of follow-up and incidental autoimmunity, unsuppressed TSH levels involved in unsuspected high absorbed-dose levels in the normal gland are to be considered first [34].

Our minute LT3 suppression test (mLT3sT) is simple and very effective in reducing TSH. Some physicians have concerns about this test, as giving additional exogenous thyroid hormone should be avoided in borderline hyperthyroid patients. However, we performed the mLT3sT on more than 1000 patients without any significant side effects. This is due to the very low dose used (25 μg/d), the physiological amount, and the short period of administration. The historical Werner’s suppression test uses 75 μg/d (LT3) for 8 days [35]. Until the 1980s, the suppression test was useful for demonstrating thyroid hypersecretion on imaging, as TSH sensitivity could reach 2.0 mU/L, a value impairing any biological evidence of hyperthyroidism. Nowadays, this diagnosis is easier due to sensitive TSH assays (1984). In our study, with a total of 125 μg of LT3 administered within 5 days, no systemic effects were observed. In cases of longstanding palpitations evidenced at baseline, a short course of beta blockers can be used until thyroid function normalizes. On the other hand, it is not certain that LT3 (Cynomel^®^, Sanofi) will still be distributed in the next few years. Hopefully, alternative LT3 capsules will be available (Thybon^®^, 20 μg per capsule, Henning), but we have occasionally used LT4 (75 μg/d for 5 days) with the same results.

The main problem with the mLT3sT was not tolerance but borderline effectiveness in a few patients who presented with TSH > 0.60 mU/L at baseline. In these patients, the suppressed qTS often suggested incomplete uptake suppression in the ENLobe, so LT3 was prolonged for up to 3 additional days.

The view that hypothyroidism after ^131^I-RIT treatment in UFA patients is rare relies upon the erroneous belief that a near-suppressed uptake in the extra-nodular tissue at imaging parallels low absorbed doses in normal tissue [34,36]. Our dosimetric calculations demonstrate the clinical outcomes of our LT3s-RIT approach, evidencing dose redistribution under LT3 suppression. A small LT3-induced decrease in TSH (mean, 0.284 mU/L) was enough to produce low residual uptake in the ENLobe (mean, 0.73%), but it had a limited effect on autonomously functioning volume uptakes, as is often reported [37]. This aligns with the small 13.5% uptake decrease in the NLobe of our patients. However, TSH stimulation can modify both iodine turnover and redistribution, as reported for rhTSH-aided ^131^I-RIT treatments of large goiters [38]. Thus, even a small TSH-stimulating value may be responsible for significant and prolonged uptake in normal extra-nodular tissue. Paralleling these locally induced uptake variations, absorbed doses were strongly reduced in the ENLobe to about 40 Gy while remaining largely unchanged in the UFA. This parallels our previous experience with AFTN patients receiving ^131^I, with or without extra-nodular visible activity [39]: in those with non-suppressed TSH levels, hypothyroidism significantly occurred in 23.8% versus 7.9%, corresponding to much higher absorbed doses in the extra-nodular lobe.

Finally, using the old “dosimetric compromise approach,” we established that an intended AD of about 130 Gy in the nodular lobe corresponds to about 260 Gy to the UFA.

All of our patients underwent the same activity calculation using the same program throughout the study. Notably, we calculated low activity levels (181 ± 98 MBq) compared with classical levels reported in UFA treated at a toxic stage. This reflects earlier treatment in smaller target volumes and the use of a personalized dosimetric approach. Administering low doses is a regulatory recommendation (EURATOM 97-43) and relevant for radioprotection [40]. Furthermore, no contact restrictions are usually needed using the LT3s-RIT protocol, except for rare and specific *scenarios*, such as sleeping next to a pregnant woman or working with babies.

To the best of our knowledge, we have demonstrated a significant absorbed-dose–effect relationship in the NLobe for the first time: the higher the absorbed dose, the higher the volume reduction.

A final objective of RIT involves nodule size reduction in patients with large AFTNs that may cause cervical discomfort. Using LT3s-RIT, UFA volume reduction was achieved at about 50%, as reported in the literature [29]. This should be compared with novel ultrasound-based techniques [41], mainly thermo-ablation (TA). While these procedures seem to offer a good alternative to surgery in common benign hypofunctioning nodules, their use in AFTNs is questionable. Indeed, they are rather expensive compared with ^131^I, with some side effects and moderately convincing results in the long term [42]. Since hypothyroidism can be avoided in many cases, these indications should be carefully weighed in the future. LT3s-RIT is likely more interesting in SCH patients with smaller UFA nodules and multifocal or disseminated variants.

When to treat is the last question. ^131^I-RIT is often thought of as an old treatment in AFTN patients, raising the risk of radioprotection issues, and is often considered for older people compared with surgery. This remains generally true when a nodule is large, suspicious upon FNA, or responsible for cervical discomfort. However, the clinical presentation evolves, and early diagnoses are routinely made in younger patients with smaller functional volumes who are likely at metabolic risk due to longstanding mild hypersecretion. As LT3s-RIT has proven to be effective, simple, and cheap, it should be compared in the near future to other modalities in SCH patients and intermediate-sized nodules.

Setting a TSH cut-off value to appropriately manage SCH is hotly debated, explaining why—according to the learned society that publishes relevant guidelines—the proposed cut-off values significantly vary with time [24,43,44,45]. This is also the case for recommendations on performing thyroid scans in the low–normal TSH range [45,46,47]. Indeed, TSH widely fluctuates in individuals over a single day. The precision of the individual TSH set point is 10% using 15 tests and only 20% using 2 tests [48]. It is difficult to believe that the same person can be hyperthyroid at 9 am but euthyroid at 3 pm. Finally, because mean TSH levels vary with geographic areas, iodine supplies, and the frequency of the underlying SCH presentation, some prefer to consider a range of TSH values suggesting hypersecretion and a decreasing slope of TSH values over time [11].

Our study has several limitations, mainly due to its retrospective design and partially available data. To better correlate clinical, biological, and dosimetric information, we selected only patients with typical UFA and a complete dataset or a prolonged follow-up. This could introduce selection bias and limit the number of eligible files. However, multifocal and disseminated variants would require specific dosimetric approaches outside the scope of this study. Another significant limitation is the absence of a control group, making comparisons possible only through published data from the literature, which may have different patient and disease patterns.

## 5. Conclusions

We report the first evidence that UFA patients can be treated early with ^131^I at subclinical hyperthyroid stage grade 1 (TSH > 0.1 mU/L), with long-term euthyroidism and low activity levels. To do so, we developed a routine minute LT3-induced procedure to suppress TSH secretion at the time of therapy (LT3s-RIT), allowing us to focus the dose on the autonomously functioning volume while largely sparing normal tissue from the irradiation. Thus, we designed a very simple protocol that only requires two outpatient visits, with excellent tolerance. LT3s-RIT is a current care protocol and uses a routine nuclear medicine procedure. We also evidenced a dose–effect relationship that fully justifies the use of personalized dosimetric tools. This could help to quickly and safely determine ^131^I activities that correspond to clinical purposes, such as normalizing thyroid function or reducing volume. Subclinical hyperthyroidism is common, underdiagnosed, and often undertreated, despite being responsible for serious clinical issues. Recent interventional studies suggest that treatment has a preventive effect on atrial fibrillation and bone loss. It is likely that a significant international prospective study to evaluate LT3s-RIT is required to confirm these preliminary findings on long-term euthyroidism maintenance and demonstrate its major clinical benefits for cardiovascular issues and bone loss as well.

## Figures and Tables

**Table 1:** Demographics and clinical status at baseline.

Baseline	All (95)	Men (*N* = 14)	Women (*N* = 81)	*p* (Mann–Whitney)
Age at 131I-RIT (y)	53.3 ± 16.3	60.5 ± 13.9	52.0 ± 16.4	0.096
Addressing TSH (mUI/L) [10th–90th]	0.35 ± 0.28 [0.10–0.43]	0.37 ± 0.27	0.34 ± 0.28	0.797
UFA Volume (mL)	5.52 ± 3.94	9.03 ± 6.37	4.90 ± 2.98	0.017
Thyroid Volume (mL)	17.45 ± 6.53	23.22 ± 7.72	16.40 ± 6.75	0.001
Delay (months) between Baseline and Suppression	3.71 ± 3.70	3.91 ± 2.71	3.65 ± 3.83	0.812
Clinical Evaluation at Baseline				
No Complaint	57/95 (60.0%)			
Palpitations	28.4%			
Atrial Fibrillation	2.1% (1 transitory, 1 permanent)			
Stroke	2.1% (1 transitory, 1 constituted)			
Osteopenia/Osteoporosis	8.9%			

^1311^I-RIT: ^131^I radioiodine therapy. UFA: unifocal autonomy (the autonomous nodule). Results are presented as mean ± SD.

**Table 2:** Evaluation of TSH suppression during LT3 administration, based on biological and quantified thyroid scan parameters.

	Baseline	Suppressed	*p*
TSH (mUI/L)	0.455 ± 0.317	0.047 ± 0.034	<0.0001
fT4 (pmol/L)	13.99 ± 2.24	13.48 ± 2.17	0.0094
fT3 (pmol/L)	5.23 ± 0.70	11.7 ± 3.27	<0.0001
Early ^123^I Uptake (120 min)	11.01 ± 3.98	8.38 ± 4.78	<0.0001
NLobe Uptake (%)	9.01 ± 3.46	7.78 ± 4.91	<0.0001
ENLobe Uptake (%)	1.77 ± 1.23	0.73 ± 0.52	<0.0001
UFA Uptake (%)	7.03 ± 2.80	4.22 ± 0.47	<0.0001
Uptake Ratio (UFA/ENLobe)	8.29 ± 13.12	20.31 ± 45.0	<0.0001
Metabolic Functional Volume ^90^	10.5 ± 4.4	9.48 ± 4.56	<0.0001

NLobe: nodular lobe; ENLobe: extra-nodular lobe; UFA: autonomously functioning nodule (unifocal autonomy). Metabolic functional volume ^90^: volume containing 90% of the administered iodine activity, according to Quanthyr; for details, see Section 2: Patients and Methods. Results are presented as mean ± SD.

**Table 3:** Dosimetric consequences of LT3-induced TSH suppression: absorbed-dose values (Gy) in the volumes of interest according to activity calculation at baseline.

Administered Activity 181 ± 98 MBq		Absorbed Dose at Baseline (Simulation)	Absorbed Dose on LT3 (Actual Values)	*p*
Mean AD (Gy) Whole Gland	All	108 ± 41	95 ± 36	<0.0001
	Low Dose	92 ± 29	84 ± 29	
	High Dose	143 ± 41	120 ± 38	
Mean AD (Gy) NLobe	All	137 ± 57	127 ± 51	<0.0001
	Low Dose	115 ± 38	113 ± 44	
	High Dose	185 ± 61	157 ± 53	
Mean AD (Gy) UFA	All	259 ± 238	257 ± 143	0.3975
	Low Dose	244 ± 125	254 ± 135	
	High Dose	292 ± 162	263 ± 160	
Mean AD (Gy) Extra-Nodular Lobe	All	61 ± 31	37 ± 20	<0.0001
	Low Dose	54 ± 23	35 ± 19	
	High Dose	75 ± 40	43 ± 22	

Activity calculations were determined at baseline to deliver two AD levels to the lobe harboring the nodule (NLobe): low dose (about 130 Gy) in 31.2%, or high dose (about 200 Gy) in 68.3% (see Patients and Methods). *p*: Wilcoxon’s test; UFA: the treated autonomous nodule. ADs (mean ± SD) are calculated using the dosimetric menu of the Quanthyr software (Planet Oncodose, release 3.3, Dosisoft (for details, see Section 2.3).

## Data Availability

The datasets presented in this article are not readily available because the data are part of ongoing studies.

## Abbreviations

^131^I	iodine ^131^I
^123^I	iodine ^123^I
Aa	therapeutic activity to be administered
AFTN	autonomously functioning thyroid nodule
B	baseline procedure
ENLobe	extra-nodular lobe
FMV^90^	functional metabolic volume
G	grade
NLobe	nodular lobe (harboring the nodule)
LT3s-RIT	^131^radioiodine therapy using a minute LT3 suppression test
mLT3SupT	minute LT3 suppression test
MPUS	multiparametric ultrasound
qTS	quantified thyroid scan
^131^I-RIT	radioiodine therapy
SCH	subclinical hyperthyroidism
S	suppressed procedure
TS	thyroid scan
UFA	unifocal autonomy
